# Supplementary material for: The effects of mindfulness-based interventions on symptoms of depression, anxiety, and cancer-related fatigue in oncology patients: A systematic review and meta-analysis
Source: PLoS One. 2022 Jul 14;17(7):e0269519. doi: 10.1371/journal.pone.0269519 (PMC9282451; doi:10.1371/journal.pone.0269519)
Supplement: S1 Table — (DOCX) [file pone.0269519.s001.docx]

| **S1 Table. Summary of the reviews undertaken in the recent ten years** | | | | | |
| --- | --- | --- | --- | --- | --- |
| Author(s) | Oncology Population | MBI(s) explored | Outcome Measures | Year of Studies Included | Types of Studies Included |
| Cramer et al.†  *k* = 2 | Breast | MBSR and MBCT | Anxiety and Depression | Prior to November 2011 | RCTs |
| Garner▪  *k* = 8 | Breast | MBSR | Anxiety and Depression | March 2009 to May 2014 | RCTs, nRCTs, Meta analysis, Qualitative Study, Literature Reviews |
| Haller et al.†  *k* = 10 | Breast | MBSR and MBCT | Anxiety, Depression, Stress, Quality of Life, Fatigue, Sleep | Prior to October 2016 | RCTs |
| Hofmann et al.†  *k* = 9 | General | MBSR and MBCT | Depression | Prior to April 2009 | RCT and Pre- and Post- Studies |
| Ledesma and Kumano†  *k* = 10 | General | MBSR | Mental and Physical Well-being | Prior to 2007 | RCT and Observational Studies |
| Piet et al.†  *k* = 22 | General | MBSR and MBCT | Anxiety and Depression | Prior to March 2012 | RCTs and nRCTs |
| Rush and Sharma  *k* = 13 | General | MBSR | Stress Reduction | October 2009 to November 2015 | RCTs and nRCTs |
| Shennan et al.  *k* = 13 | General | MBSR, MBCT, Meditation | Anxiety, Depression, Stress, Sexual Difficulties, Physiological Arousal, and Immune Function | January 2000 to September 2009 | RCT, Pre- and Post- Studies, Qualitative Studies |
| Zainal et al.†  *k* = 9 | Breast | MBSR | Anxiety, Depression, Stress | Prior to November 2011 | RCTs and nRCTs |
| Zhang et al.†  *k* = 7 | General | MBSR, Mindfulness-based art therapy | Anxiety and Depression | Prior to November 2014 | RCTs |
| MBI(s) explored include(s) the modified version of the original MBSR and MBCT, which tailored the treatment duration to suit the need of the cancer population. *k =* number of studies that were included in the review; MBSR = Mindfulness-Based Stress Reduction; MBCT = Mindfulness-Based Cognitive Therapy; RCT = Randomised Controlled Trial; nRCT = Non-Randomised Controlled Trial.  †indicates meta-analysis.  ▪ indicates thesis. | | | | | |
