# Supplementary material for: The effects of mindfulness-based interventions on symptoms of depression, anxiety, and cancer-related fatigue in oncology patients: A systematic review and meta-analysis
Source: PLoS One. 2022 Jul 14;17(7):e0269519. doi: 10.1371/journal.pone.0269519 (PMC9282451; doi:10.1371/journal.pone.0269519)
Supplement: S2 Table — (DOCX) [file pone.0269519.s002.docx]

| **S2 Table. Summary of MBI in each study** | | | | | | | | | | | |
| --- | --- | --- | --- | --- | --- | --- | --- | --- | --- | --- | --- |
| Study (Year) | Oncology Population | | Participants | | Interventions | | | | | | |
|  | Site | Stages | Mean age (SD) | % Female | MBI | No. of Sessions | Duration of Sessionꚜ | Retreat | Yoga | Home-workꚜ | Total Contact Hours |
| Non-Randomised Controlled Studies | | | | | | | | | | | |
| Birnie et al. (2010) | Mixed | All | 62.9 (7.4) | 52.4 | MBSR | 8 | 90 | 3 | √ | 45-60 | 15 |
| Carlson et al. (2001) | Mixed | 0-IV | 52.0 (10.5) | 80.9 | MBSR | 7 | 90 | 0 | √ | 40-45 | 10.5 |
| Carlson et al. (2003) | Breast & Prostate | 0-III | 54.5 (10.9) | 78.6 | MBSR | 8 | 90 | 3 |  | 40 | 15 |
| Carlson & Garland (2005) | Mixed | All | 54.0 (-) | 77.8 | MBSR | 7 | 90 | 3 | √ | 45 | 13.5 |
| Chambers et al. (2012) | Prostate | Advanced | 67.0 (6.5) | 0 | MBCT | 8 | 120 | 0 | √ | 35 | 16 |
| Dobkin (2008) | Breast | All | 54.0 (-) | 100.0 | MBSR | 8 | 120 | 0 | √ | 45-60 | 16 |
| Dobos et al. (2015) | Mixed | Survivors | 53.9 (10.7) | 91.0 | MBSR | 11 | 360 | 0 | √ | - | 66 |
| Eyles et al. (2015) | Breast | Metastatic | - | 100.0 | MBSR | 7 | 120 | 5.5 | √ | 30 | 19.5 |
| Garland et al. (2007) | Mixed | All | 52.2 (-) | 90.0 | MBSR | 8 | 90 | 3 | √ | 45 | 15 |
| Garland et al. (2013) | Mixed | All | 53.5 (10.6) | 84.0 | MBCR | 8 | 90 | 6 | √ | 45 | 18 |
| Johns et al. (2020) | Mixed | III-IV | 62.9 (10.6) | 46.2 | MBSR | 6 | 120 | 0 | √ | 20 | 12 |
| Kieviet-Stijnen et al. (2008) | Mixed | All | 48.4 (7.6) | 72.0 | MBSR | 7 | 150 | 6 | √ | 45 | 23.5 |
| Labelle et al. (2010) | Mixed | All | 53.1 (8.9) | 100.0 | MBCR | 8 | 90 | 6 | √ | 45 | 18 |
| Lee et al. (2017) | Breast | Metastatic | - | 100.0 | MBSR | 8 | 120 | 0 | √ | 50 | 16 |
| Lengacher et al. (2011) | Breast | 0-III | 56.8 (8.8) | 100.0 | MBSR | 8 | 120 | 0 | √ | 45 | 16 |
| Matousek & Dobkin (2010) | Breast | All | 56.4 (10.2) | 100.0 | MBSR | 8 | 150 | 6 | √ | 45-60 | 26 |
| Park et al. (2018) | Breast | I-III | 50.1 (9.1) | 100.0 | MBCT | 8 | 120 | 0 |  | 20-45 | 16 |
| Rahmani et al. (2014) | Breast | I-III | 43.3 (3.1) | 100.0 | MBSR | 8 | 120 | 0 |  | 45 | 16 |
| Tacon et al. (2004) | Breast | All | 53.3 (-) | 100.0 | MBSR | 8 | 90 | 0 | √ | 45 | 12 |
| van den Hurk et al. (2015) | Lung | All | 61.7 (-) | 47.0 | MBSR | 8 | 150 | 6 | √ | 45 | 26 |
| Randomised Controlled Studies | | | | | | | | | | | |
| Foley et al. (2010) | Mixed | All | 55.2 (10.6) | 77.0 | MBCT | 8 | 120 | 5 |  | 40-60 | 21 |
| Hoffman et al. (2012) | Breast | 0-III | 49.0 (9.3) | 100.0 | MBSR | 7 | 120 | 6 | √ | 40-45 | 20 |
| Johns et al. (2015) | Mixed | All | 58.8 (9.3) | 94.0 | MBSR | 7 | 120 | 0 | √ | 20 | 14 |
| Johns et al. (2016) | Breast & Colorectal | Survivors | 56.9 (9.9) | 94.3 | MBSR | 8 | 120 | 0 |  | 20 | 16 |
| Kenne Sarenmalm et al. (2017) | Breast | All | 57.2 (10.2) | 100.0 | MBSR | 8 | 120 | 0 | √ | 20 | 16 |
| Kingston et al. (2015) | Mixed | In remission | 49.8 (10.9) | 50.0 | MBCT | 8 | 90 | 0 |  | - | 12 |
| Lengacher et al. (2012) | Breast | 0-III survivor | 57.5 (9.4) | 100.0 | MBSR | 6 | 120 | 0 | √ | 15-45 | 12 |
| Lengacher et al. (2014) | Breast | 0-III survivor | 57.2 (9.2) | 100.0 | MBSR | 6 | 120 | 0 | √ | 15-45 | 12 |
| Lengacher et al. (2016) | Breast | Survivors | 56.5 (10.2) | 100.0 | MBSR | 6 | 120 | 0 | √ | 15-45 | 12 |
| Liu et al. (2019) | Thyroid | 0-IV | 43.3 (11.0) | 69.4 | MBSR | 8 | 120 | 0 | √ | - | 16 |
| Meiklejon (2008) | Breast | 0-III | 55.0 (9.5) | 100.0 | MBSR | 6 | 90 | 0 |  | - | 9 |
| Pouy et al. (2018) | Breast | 0-III | 52.1 (11.1) | 100.0 | MBSR | 8 | 90 | 0 | √ | - | 12 |
| Speca et al. (2000) | Mixed | 0-IV | 54.9 (10.5) | 85.3 | MBSR | 7 | 90 | 0 | √ | 40-45 | 10.5 |
| van der Lee & Garssen (2012) | Mixed | All | 53.1 (9.1) | 86.0 | MBCT | 8 | 150 | 6 |  | - | 26 |
| Witek Janusek & Mathews (2019) | Breast | Early | 55 (10.1) | 100.0 | MBSR | 7 | 180 | 6 | √ | 40-45 | 27 |
| Zhang et al. (2017) | Breast | I-III | 48.7 (8.5) | 100.0 | MBSR | 8 | 120 | 0 | √ | 40-45 | 16 |
| MBSR = Mindfulness-Based Stress Reduction; MBCT = Mindfulness-Based Cognitive Therapy; RCT = Randomised Controlled Trial; nRCT = Non-Randomised Controlled Trial.  Dashes (-) indicate that the specified information was not reported in the study.  ꚜ = minutes. | | | | | | | | | | | |
