# Supplementary material for: The effects of mindfulness-based interventions on symptoms of depression, anxiety, and cancer-related fatigue in oncology patients: A systematic review and meta-analysis
Source: PLoS One. 2022 Jul 14;17(7):e0269519. doi: 10.1371/journal.pone.0269519 (PMC9282451; doi:10.1371/journal.pone.0269519)
Supplement: S3 Table — (DOCX) [file pone.0269519.s003.docx]

| **S3 Table. Within group effect sizes for pre- and post-intervention scores** | | | | | | | | | | |
| --- | --- | --- | --- | --- | --- | --- | --- | --- | --- | --- |
| Study (Year) | MBI | Anxiety | | | Depression | | | CRF | | |
|  |  | Measure | Unbiased Hedges’ *g* | 95% CI | Measure | Unbiased Hedges’ *g* | 95% CI | Measure | Unbiased Hedges’ *g* | 95% CI |
| Non-Randomised Controlled Studies | | | | | | | | | | |
| Birnie et al. (2010) | MBSR | POMS | 0.39 | [-0.22, 1.00] | POMS | 0.28 | [-0.33, 0.89] | POMS | 0.40 | [-0.20, 1.02] |
| Carlson et al. (2001) | MBSR | POMS | 0.61 | [0.25, 0.97] | POMS | 0.50 | [0.08, 0.80] | POMS | 0.30 | [-0.01, 0.60] |
| Carlson et al. (2003) | MBSR | POMS | 0.64 | [0.34, 0.96] | POMS | 0.02 | [-0.40, 0.45] | POMS | -1.08 | [-1.55, -0.63] |
| Carlson & Garland (2005) | MBSR | POMS | 0.10 | [-0.33, 0.53] | POMS | 0.44 | [0.08, 0.80] | POMS | 0.38 | [0.03, 0.74] |
| Chambers et al. (2012) | MBCT | HADS-A | 0.33 | [-0.47, 1.15] | HADS-A | 0.13 | [-0.67, 0.93] | **-** | **-** | **-** |
| Dobkin (2008) | MBSR | **-** | **-** | **-** | CESD | 0.63 | [-0.14, 1.44] | **-** | **-** | **-** |
| Dobos et al. (2015) | MBSR | HADS-A | 0.60 | [0.33, 0.87] | HADS-D | 0.55 | [0.28, 0.82] | EORTC QLQ-C30 | 0.31 | [0.04, 0.58] |
| Eyles et al. (2015) | MBSR | HADS-A | 0.76 | [0.12, 1.43] | HADS-D | 0.16 | [-0.48, 0.80] | BFI | 0.14 | [-0.50, 0.77] |
| Garland et al. (2007) | MBSR | POMS | 0.49 | [0.13, 0.86] | POMS | 0.38 | [0.02, 0.75] | POMS | 0.29 | [-0.07, 0.65] |
| Garland et al. (2013) | MBCR | POMS | 0.51 | [0.22, 0.81] | POMS | 0.43 | [0.13, 0.72] | POMS | 0.37 | [0.08, 0.66] |
| Johns et al. (2020) | MBSR | GAD-7 | 0.71 | [-0.06, 1.53] | PHQ-8 | 0.51 | [-0.25, 1.31] | FSI | -0.01 | [-0.78, 0.76] |
| Kieviet-Stijnen et al. (2008) | MBSR | POMS | 0.39 | [-0.02, 0.80] | POMS | 0.30 | [-0.10, 0.71] | POMS | 0.11 | [-0.29, 0.52] |
| Labelle et al. (2010) | MBCR | **-** | **-** | **-** | CES-D | 0.55 | [0.14, 0.97] | **-** | **-** | **-** |
| Lee et al. (2017) | MBSR | HADS-A | 0.15 | [-0.80, 1.08] | HADS-D | 0.37 | [-0.55, 1.32] | **-** | **-** | **-** |
| Lengacher et al. (2011) | MBSR | STAI | 0.48 | [-0.19, 1.18] | CES-D | 0.75 | [0.07, 1.46] | QF-36 Health Survey | -0.38 | [-1.07, 0.29] |
| Matousek & Dobkin (2010) | MBSR | **-** | **-** | **-** | CES-D | 0.68 | [0.31, 1.06] | **-** | **-** | **-** |
| Park et al. (2018) | MBCT | HADS-A | 0.74 | [-0.06, 1.60] | HADS-D | 0.47 | [-0.33, 1.29] | **-** | **-** | **-** |
| Rahmani et al. (2014) | MBSR | **-** | **-** | **-** | **-** | **-** | **-** | QLQ-C30 | 5.44 | [3.85, 7.41] |
| Tacon et al. (2004) | MBSR | STAI | 1.37 | [0.79, 1.98] | **-** | **-** | **-** | **-** | **-** | **-** |
| van den Hurk et al. (2015) | MBSR | HADS-A | 0.34 | [-0.42, 1.13] | HADS-D | -0.07 | [-0.84, 0.69] | CSI-Fatigue | 0.17 | [-0.59, 0.95] |
| Randomised Controlled Studies | | | | | | | | | | |
| Foley et al. (2010) | MBCT | HAM-A | 1.38 | [0.97, 1.81] | HAM-D | 1.51 | [1.095, 1.94] | **-** | **-** | **-** |
| Hoffman et al. (2012) | MBSR | POMS | 0.40 | [0.12, 0.68] | POMS | 0.27 | [-0.01, 0.54] | POMS | 0.38 | [0.11, 0.66] |
| Johns et al. (2015) | MBSR | GAD-7 | 0.69 | [0.03, 1.38] | PHQ-8 | 0.77 | [0.11, 1.47] | FSI | 1.26 | [0.57, 2.01] |
| Johns et al. (2016) | MBSR | GAD-7 | 0.89 | [0.41, 1.40] | PHQ-8 | 1.04 | [0.55, 1.56] | FSI | 0.85 | [0.36, 1.35] |
| Kenne Sarenmalm et al. (2017) | MBSR | HADS-A | 0.12 | [-0.23, 0.47] | HADS-D | 0.28 | [-0.07, 0.64] | **-** | **-** | **-** |
| Kingston et al. (2015) | MBCT | HADS-A | 0.54 | [-0.23, 1.34] | HADS-D | -0.48 | [-1.28, 0.28] | **-** | **-** | **-** |
| Lengacher et al. (2016) | MBSR | STAI | 0.64 | [0.42, 0.86] | CES-D | 0.44 | [0.22, 0.66] | FSI | 0.49 | [0.27, 0.71] |
| Lengacher et al. (2012) | MBSR | **-** | **-** | **-** | **-** | **-** | **-** | MDSAI | 0.64 | [0.20, 1.10] |
| Lengacher et al. (2014) | MBSR | STAI | 0.71 | [0.26, 1.16] | CES-D | 0.67 | [0.22, 1.12] | **-** | **-** | **-** |
| Liu et al. (2019) | MBSR | SAS | -0.54 | [-0.95, -0.14] | SDS | -0.75 | [-1.16, -0.34] | EORTC QLQ-C30 | -1.26 | [-1.70, -0.83] |
| Meiklejon (2008 | MBSR | **-** | **-** | **-** | POMS | 0.40 | [-0.12, 0.92] | POMS | 0.61 | [0.08, 1.14] |
| Pouy et al. (2018) | MBSR | DASS-21 | 1.35 | [0.82, 1.91] | DASS-21 | 1.11 | [0.60, 1.65] | **-** | **-** | **-** |
| Speca et al. (2000) | MBSR | POMS | 0.69 | [0.30, 1.08] | POMS | 0.58 | [0.20, 0.97] | POMS | 0.43 | [0.05, 0.82] |
| van der Lee & Garssen (2012) | MBCT | **-** | **-** | **-** | **-** | **-** | **-** | CIS-Fatigue | 1.30 | [0.91, 1.71] |
| Witek Janusek & Mathews (2019) | MBSR | **-** | **-** | **-** | CES-D | 0.29 | [-0.02, 0.61] | MSFI-SF | 0.10 | [-0.22, 0.42] |
| Zhang et al. (2017) | MBSR | STAI | 0.80 | [0.27, 1.36] | **-** | **-** | **-** | **-** | **-** | **-** |
| Hedges’ g was calculated with the standardised mean difference between pre-intervention scores and post-intervention scores (footnote of page 13). BDI = Beck’s Depressive Inventory; BFI = Brief Fatigue Inventory; CRF = cancer-related fatigue; CES-D = Center for Epidemiologic Studies, Depression Scale; CIS = Checklist Individual Strength; DASS-21 = Depression, Anxiety and Stress Scale; EORTC-QOQ-C30 = European Organisation for Research and Treatment of Cancer Quality of Life Questionnaire; FSI = Fatigue Symptom Inventory; GAD‐7 = seven‐item Patient Health Questionnaire Generalized Anxiety Disorder Scale; HADS = Hospital Anxiety and Depression Scale; MDASI = M.D. Anderson Symptom Inventory; MFSI-SF = Multidimensional Fatigue Scale Inventory =Short Form; PHQ‐8 = Patient Health Questionnaire eight‐item depression scale; POMS = Profile of Mood States; SAS = Self-rating Anxiety Scale; SCL-90-R –=Symptoms Check List Revised; SDS = Self-rating Depression Scale; STAI = State-Trait Anxiety Inventory.  Dashes (-) indicate that the specified information was not reported in the study. | | | | | | | | | | |
