# Supplementary material for: The effects of mindfulness-based interventions on symptoms of depression, anxiety, and cancer-related fatigue in oncology patients: A systematic review and meta-analysis
Source: PLoS One. 2022 Jul 14;17(7):e0269519. doi: 10.1371/journal.pone.0269519 (PMC9282451; doi:10.1371/journal.pone.0269519)
Supplement: S4 Table — (DOCX) [file pone.0269519.s004.docx]

| **S4 Table. Longer term within group effect sizes** | | | | | | | | | | | |
| --- | --- | --- | --- | --- | --- | --- | --- | --- | --- | --- | --- |
| Study (Year) | Period (months) | Anxiety | | | Depression | | | CRF | | | |
|  |  | Measure | Unbiased Hedges’ *g* | 95% CI | Measure | Unbiased Hedges’ *g* | 95% CI | Measure | Unbiased Hedges’ *g* | 95% CI | |
| Non-Randomised Controlled Studies | | | | | | | | | | | |
| Carlson et al. (2001) | 6 | POMS | 0.66 | [0.32, 1.01] | POMS | 0.66 | [0.31, 1.01] | POMS | 0.54 | [0.20, 0.88] | |
| Chambers et al. (2012) | 3 | HADS-A | 0.73 | [-0.075, 1.58] | HADS-A | 0.20 | [-0.60, 1.01] | **-** | **-** | **-** | |
| Dobos et al. (2015) | 3 | HADS-A | 0.55 | [0.28, 0.82] | HADS-D | 0.49 | [0.21, 0.76] | EORTC QLQ-C30 | 0.35 | [0.077, 0.62] | |
| Eyles et al. (2015) | 4 | HADS-A | 1.06 | [0.40, 1.76] | HADS-D | 0.59 | [-0.043, 1.26] | BFI | 0.42 | [-0.21, 1.08] | |
| Kieviet-Stijnen et al. (2008) | 12 | POMS | 0.68 | [0.27, 1.10] | POMS | 0.57 | [0.16, 0.98] | POMS | 0.25 | [-0.16, 0.65] | |
| Park et al. (2018) | 3 | HADS-A | 0.74 | [-0.06, 1.60] | HADS-D | 0.72 | [-0.085, 1.57] | **-** | **-** | **-** | |
| van den Hurk et al. (2015) | 3 | HADS-A | 0.47 | [-0.37, 1.36] | HADS-D | 0.0 | [-0.85, 0.85] | CSI-Fatigue | 0.078 | [-0.77, 0.93] | |
| Randomised Controlled Studies | | | | | | | | | | |  |
| Foley et al. (2010) | 3 | HAM-A | 1.44 | [1.03, 1.87] | HAM-D | 1.60 | [1.18, 2.04] | **-** | **-** | **-** | |
| Hoffman et al. (2012) | 3 | POMS | 0.40 | [0.12, 0.67] | POMS | 0.23 | [-0.041, 0.51] | POMS | 0.28 | [0.01, 0.56] | |
| Johns et al. (2016) | 6 | GAD-7 | 0.69 | [0.21, 1.19] | PHQ-8 | 0.92 | [0.43, 1.43] | FSI | 0.55 | [0.068, 1.04] | |
| Kingston et al. (2015) | 3 | HADS-A | 1.54 | [0.71, 2.47] | HADS-D | 2.67 | [1.687, 3.84] | **-** | **-** | **-** | |
| Lengacher et al. (2016) | 3 | STAI | 0.56 | [0.33, 0.78] | CES-D | 0.33 | [0.115, 0.56] | FSI | 0.48 | [0.26, 0.70] | |
| Liu et al. (2019) | 3 | SAS | 1.08 | [0.67, 1.52] | SDS | 0.87 | [0.46, 1.29] | EORTC QLQ-C30 | -0.21 | [-0.61, 0.19] | |
| Meiklejon (2008) | 3 | **-** | **-** | **-** | POMS | 0.57 | [0.04, 1.09] | POMS | 0.83 | [0.29, 1.37] | |
| van der Lee & Garssen (2012) | 6 | **-** | **-** | **-** | **-** | **-** | **-** | CIS-Fatigue | 1.30 | [0.91, 1.71] | |
| Witek Janusek & Mathews (2019) | 6 | **-** | **-** | **-** | CES-D | 0.29 | [-0.034, 0.62] | MSFI-SF | 0.35 | [0.02, 0.68] | |
| Zhang et al. (2017) | 3 | STAI | 1.19 | [0.63, 1.77] | **-** | **-** | **-** | **-** | **-** | **-** | |
| Hedges’ *g* was calculated with the standardised mean difference of the scores between the last available follow-up data and pre-intervention. BDI = Beck’s Depressive Inventory; BFI = Brief Fatigue Inventory; CES-D = Center for Epidemiologic Studies, Depression Scale; CIS = Checklist Individual Strength; DASS-21 = Depression, Anxiety and Stress Scale; EORTC-QOQ-C30 = European Organisation for Research and Treatment of Cancer Quality of Life Questionnaire; FSI = Fatigue Symptom Inventory; GAD‐7 = seven‐item Patient Health Questionnaire Generalized Anxiety Disorder Scale; HADS = Hospital Anxiety and Depression Scale; MDASI = M.D. Anderson Symptom Inventory; MFSI-SF = Multidimensional Fatigue Scale Inventory = Short Form; PHQ‐8 = Patient Health Questionnaire eight‐item depression scale; POMS = Profile of Mood States; SAS = Self-rating Anxiety Scale; SCL-90-R = Symptoms Check List Revised; SDS = Self-rating Depression Scale; STAI = State-Trait Anxiety Inventory. | | | | | | | | | | | |
