# Supplementary material for: The effects of mindfulness-based interventions on symptoms of depression, anxiety, and cancer-related fatigue in oncology patients: A systematic review and meta-analysis
Source: PLoS One. 2022 Jul 14;17(7):e0269519. doi: 10.1371/journal.pone.0269519 (PMC9282451; doi:10.1371/journal.pone.0269519)
Supplement: S5 Table — (DOCX) [file pone.0269519.s005.docx]

**S5 Table. Between group effect sizes comparing MBIs to control condition**

| Study | MBI | Anxiety | | | Depression | | | CRF | | |
| --- | --- | --- | --- | --- | --- | --- | --- | --- | --- | --- |
|  |  | Measure | Unbiased Hedges’ *g* | 95% CI | Measure | Unbiased Hedges’ *g* | 95% CI | Measure | Unbiased Hedges’ *g* | 95% CI |
| Foley et al. (2010) | MBCT | HAM-A | 0.54 | [0.17, 0.91] | HAM-D | 0.79 | [0.42, 1.18] | - | - | - |
| Hoffman et al. (2012) | MBSR | POMS | 0.38 | [0.11, 0.65] | POMS | 0.17 | [-0.09, 0.44] | POMS | 0.34 | [0.07, 0.61] |
| Johns et al. (2015) | MBSR | GAD-7 | 0.28 | [-0.37, 0.95] | PHQ-8 | 0.13 | [-0.52, 0.8] | FSI | 1.69 | [0.95, 2.5] |
| Johns et al. (2016) | MBSR | GAD-7 | 0.2 | [-0.27, 0.67] | PHQ-8 | 0.07 | [-0.39, 0.54] | FSI | 0.05 | [-0.42, 0.52] |
| Kenne Sarenmalm et al. (2017) | MBSR | HADS-A | 0.29 | [-0.07, 0.67] | HADS-D | 0.36 | [0.00, 0.73] | - | - | - |
| Kingston et al. (2015) | MBCT | HADS-A | 1.2 | [0.10, 2.50] | HADS-D | -0.36 | [-1.49, 0.7] | - | - | - |
| Lengacher et al. (2012) | MBSR | - | - | - | - | - | - | MDSAI | 0.38 | [-0.04, 0.81] |
| Lengacher et al. (2014) | MBSR | STAI | 0.12 | [-0.31, 0.55] | CES-D | 0.41 | [-0.02, 0.85] | - | - | - |
| Lengacher et al. (2016) | MBSR | STAI | 0.31 | [0.09, 0.53] | CES-D | 0.24 | [0.01, 0.47] | FSI | 0.35 | [0.12, 0.58] |
| Meiklejon (2008) | MBSR | **-** | **-** | **-** | POMS | -0.56 | [-1.14, 0.02] | POMS | -0.79 | [-1.37, -0.20] |
| Liu et al. (2019) | MBSR | SAS | 1.14 | [0.73, 1.57] | SDS | 1.23 | [0.82, 1.67] | EORTC QLQ-C30 | 1.21 | [0.79, 1.64] |
| Pouy et al. (2018) | MBSR | DASS-21 | 1.18 | [0.67, 1.72] | DASS-21 | 0.91 | [0.42, 1.43] | - | - | - |
| Rahmani et al. (2014) | MBSR | **-** | - | - | - | - | - | EORTC QLQ-C30 | 3.81 | [2.57, 5.33] |
| Speca et al. (2000) | MBSR | POMS | 0.64 | [0.21, 1.07] | POMS | 0.55 | [0.12, 0.98] | POMS | -0.01 | [-0.78, 0.76] |
| van der Lee & Garssen (2012) | MBCT | **-** | - | - | - | - | - | CIS-Fatigue | 0.92 | [0.43, 1.42] |
| Witek Janusek & Mathews (2019) | MBSR | **-** | - | - | CES-D | 1.01 | [0.47, 1.57] | MSFI-SF | -0.01 | [-0.34, 0.32] |
| Zhang et al. (2017) | MBSR | STAI | 0.65 | [0.13, 1.18] | - | - | - | - | - | - |
| Hedges’ *g* was calculated with the standardised mean difference between scores of treatment group and control group. The precise formula used can be found in the footnote of page 14. BDI = Beck’s Depressive Inventory; BFI = Brief Fatigue Inventory; CES-D = Center for Epidemiologic Studies, Depression Scale; CIS = Checklist Individual Strength; DASS-21 = Depression, Anxiety and Stress Scale; EORTC-QLQ-C30 = European Organisation for Research and Treatment of Cancer Quality of Life Questionnaire; FSI = Fatigue Symptom Inventory; GAD‐7 = seven‐item Patient Health Questionnaire Generalized Anxiety Disorder Scale; HADS = Hospital Anxiety and Depression Scale; MDASI = M.D. Anderson Symptom Inventory; MFSI-SF = Multidimensional Fatigue Scale Inventory = Short Form; PHQ‐8 = Patient Health Questionnaire eight‐item depression scale; POMS = Profile of Mood States; SAS = Self-rating Anxiety Scale; SCL-90-R = Symptoms Check List Revised; SDS = Self-rating Depression Scale; STAI = State-Trait Anxiety Inventory.  Dashes (-) in the table indicate that the specified outcome variable was not explored in the study. | | | | | | | | | | |
