# Supplementary material for: The effects of mindfulness-based interventions on symptoms of depression, anxiety, and cancer-related fatigue in oncology patients: A systematic review and meta-analysis
Source: PLoS One. 2022 Jul 14;17(7):e0269519. doi: 10.1371/journal.pone.0269519 (PMC9282451; doi:10.1371/journal.pone.0269519)
Supplement: S6 Table — (DOCX) [file pone.0269519.s006.docx]

**S6 Table. Longer term between group effect sizes of MBIs compared to control condition**

| Study | Period (months) | Anxiety | | | Depression | | | CRF | | |
| --- | --- | --- | --- | --- | --- | --- | --- | --- | --- | --- |
|  |  | Measure | Unbiased Hedges’ *g* | 95% CI | Measure | Unbiased Hedges’ *g* | 95% CI | Measure | Unbiased Hedges’ *g* | 95% CI |
| Hoffman et al. (2012) | 3 | POMS | 0.30 | [0.03, 0.57] | POMS | 0.07 | [-0.19, 0.34] | POMS | 0.22 | [-0.04, 0.49] |
| Johns et al. (2016) | 6 | GAD-7 | 0.14 | [-0.32, 0.62] | PHQ-8 | 0.14 | [-0.33, 0.62] | FSI | -0.35 | [-0.83, 0.12] |
| Lengacher et al. (2016) | 3 | STAI | 0.31 | [0.09, 0.54] | CES-D | 0.25 | [0.03, 0.48] | FSI | 0.33 | [0.11, 0.56] |
| Liu et al. (2019) | 3 | SAS | 1.25 | [0.83, 1.68] | SDS | 1.56 | [1.13, 2.02] | EORTC QLQ-C30 | 0.67 | [0.27, 1.07] |
| Witek Janusek & Mathews (2019) | 6 | **-** | **-** | **-** | CES-D | 0.00 | [-0.35, 0.34] | MSFI-SF | 0.17 | [-0.17, 0.52] |
| Zhang et al. (2017) | 3 | STAI | 1.00 | [0.47, 1.56] | **-** | **-** | **-** | **-** | **-** | **-** |
| Hedges’ *g* was calculated with the standardised mean difference between scores of treatment group and control group at least 3 months post intervention. The precise formula used can be found in the footnote of page 14. CES-D = Center for Epidemiologic Studies, Depression Scale; EORTC-QOQ-C30 = European Organisation for Research and Treatment of Cancer Quality of Life Questionnaire; FSI = Fatigue Symptom Inventory; GAD‐7 = seven‐item Patient Health Questionnaire Generalized Anxiety Disorder Scale; MFSI-SF = Multidimensional Fatigue Scale Inventory = Short Form; POMS = Profile of Mood States; SAS = Self-rating Anxiety Scale; SDS = Self-rating Depression Scale; STAI = State-Trait Anxiety Inventory.  Dashes (-) in the table indicate that the specified outcome variable was not explored in the study. | | | | | | | | | | |
