# Supplementary figures and images for: The effects of mindfulness-based interventions on symptoms of depression, anxiety, and cancer-related fatigue in oncology patients: A systematic review and meta-analysis
Source: PLoS One. 2022 Jul 14;17(7):e0269519. doi: 10.1371/journal.pone.0269519 (PMC9282451; doi:10.1371/journal.pone.0269519)

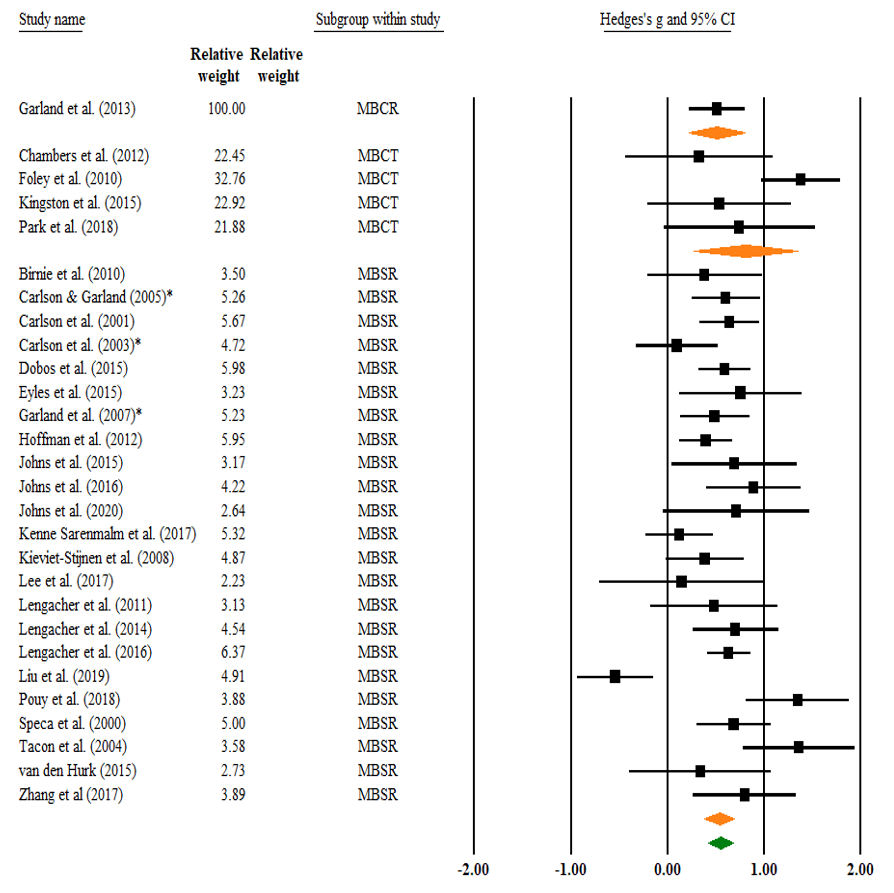

Supplement: S1 Fig — (TIF) [file pone.0269519.s007.tif]

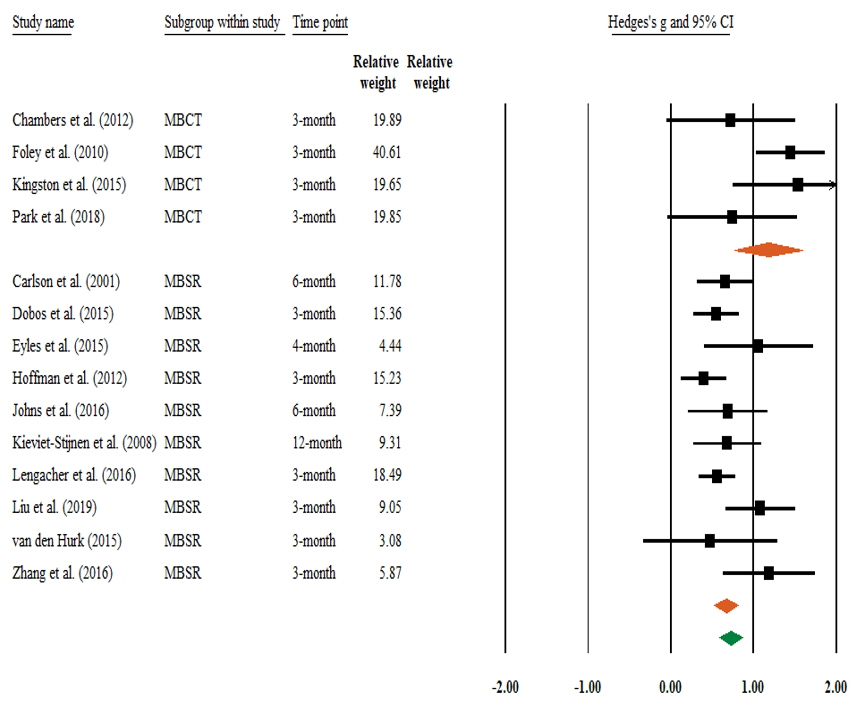

Supplement: S2 Fig — (TIF) [file pone.0269519.s008.tif]

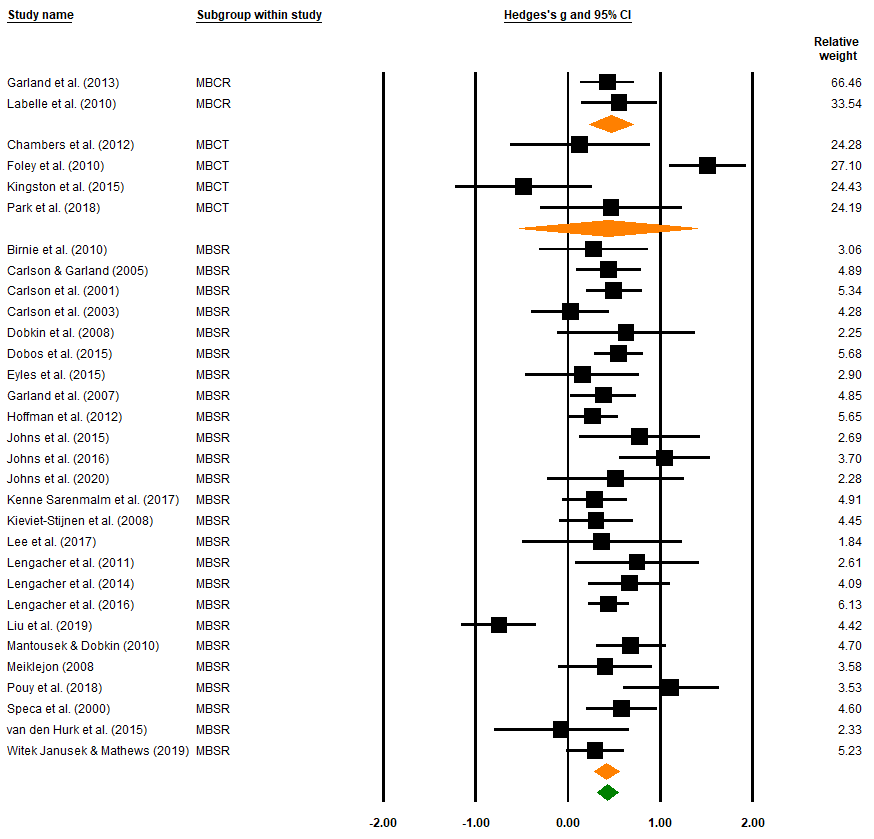

Supplement: S3 Fig — (TIF) [file pone.0269519.s009.tif]

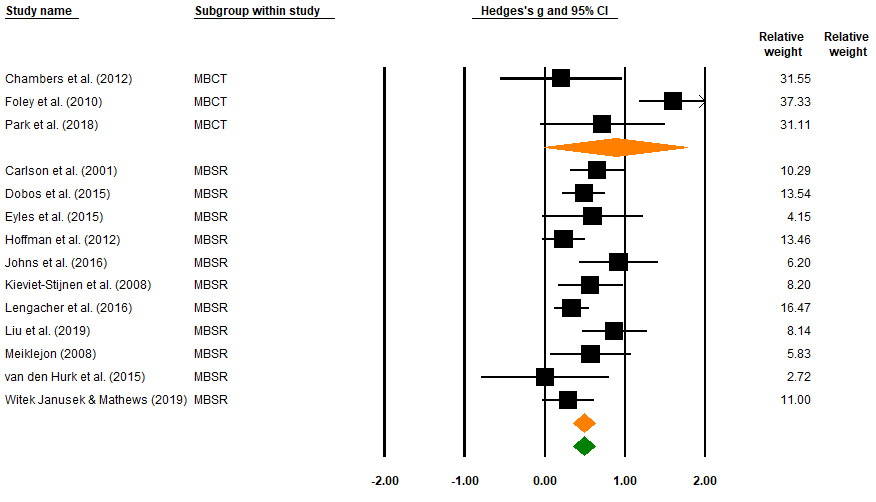

Supplement: S4 Fig — (TIF) [file pone.0269519.s010.tif]

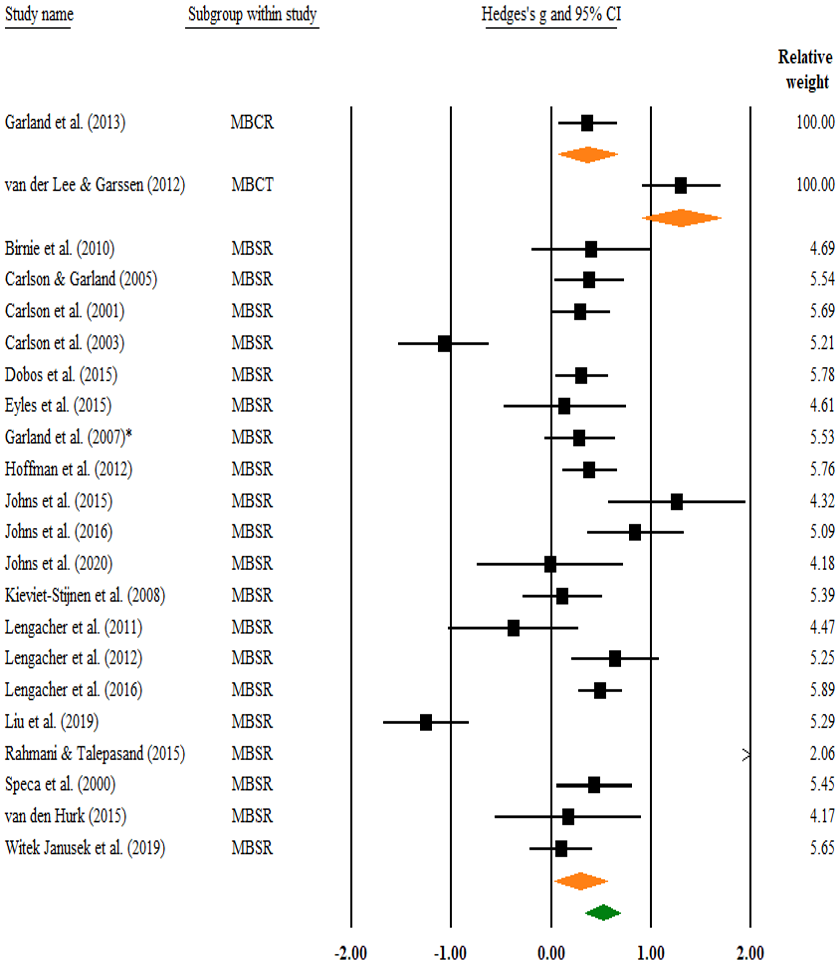

Supplement: S5 Fig — (PNG) [file pone.0269519.s011.PNG]

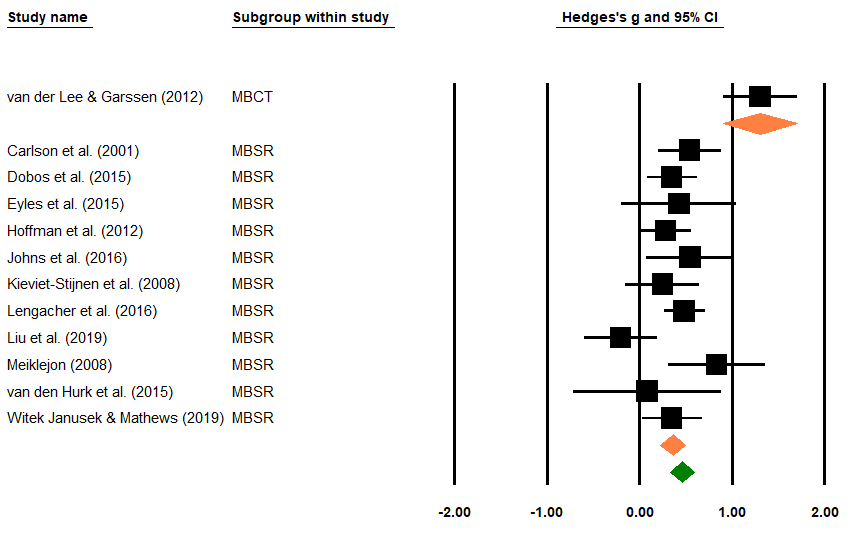

Supplement: S6 Fig — (TIF) [file pone.0269519.s012.tif]

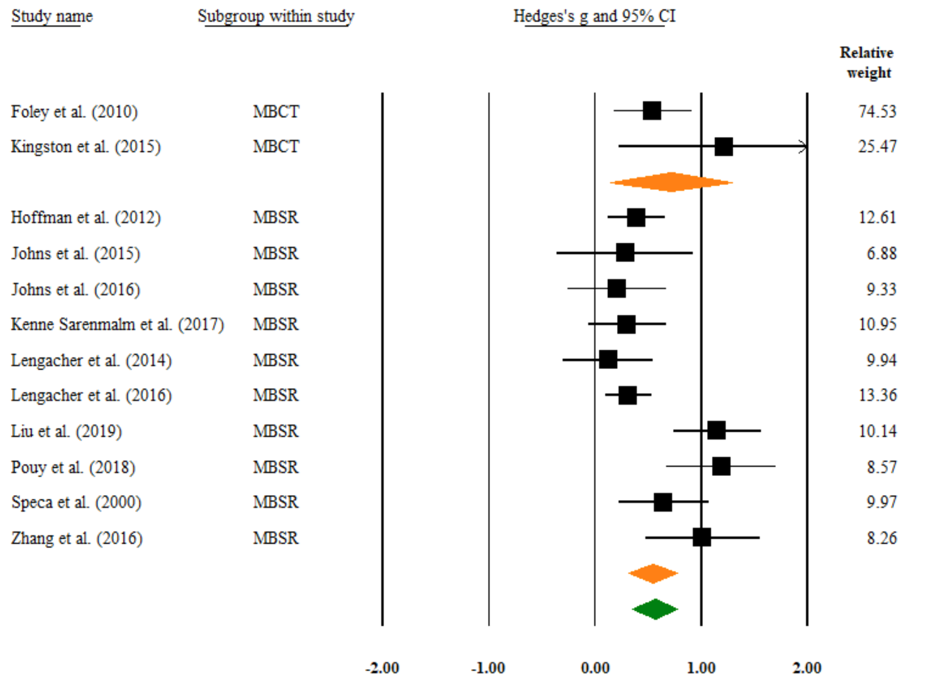

Supplement: S7 Fig — (TIF) [file pone.0269519.s013.tif]

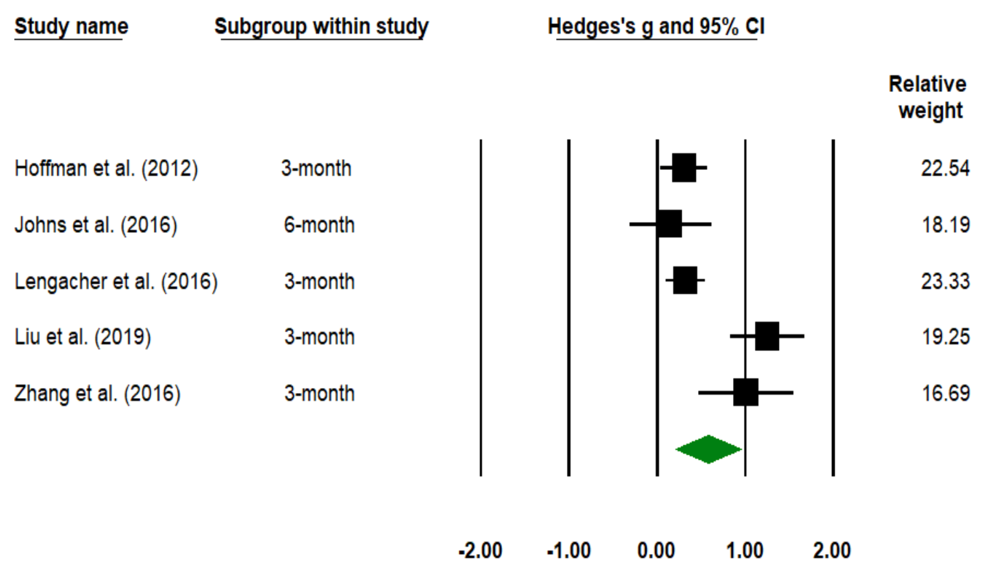

Supplement: S8 Fig — (TIF) [file pone.0269519.s014.tif]

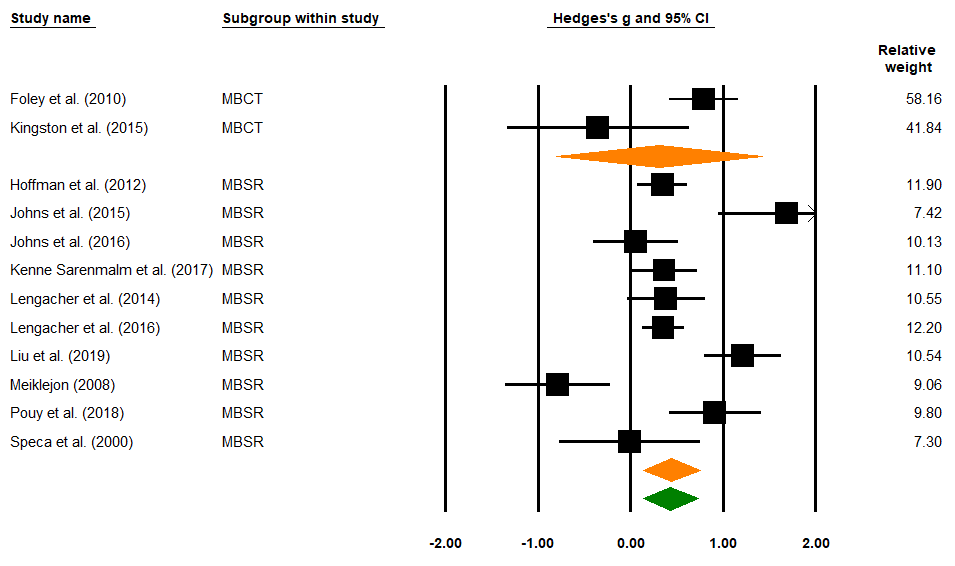

Supplement: S9 Fig — (TIF) [file pone.0269519.s015.tif]

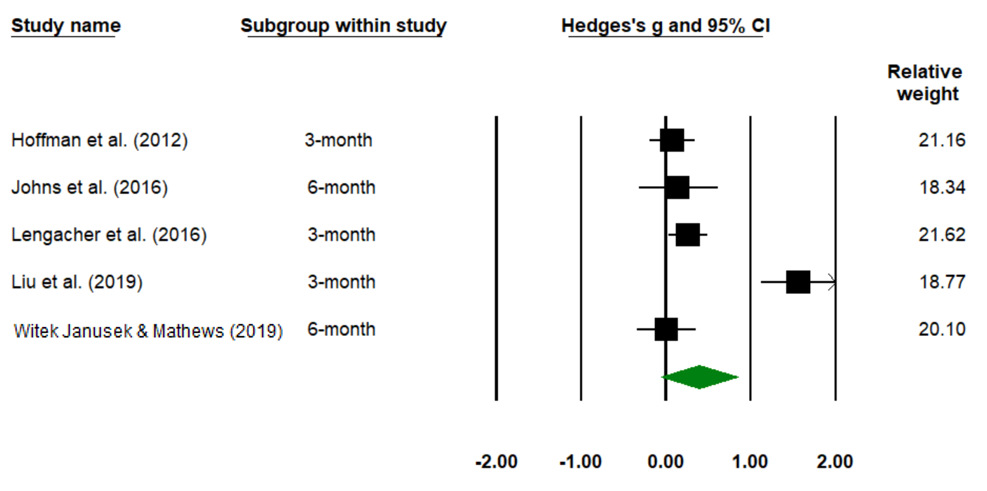

Supplement: S10 Fig — (TIF) [file pone.0269519.s016.tif]

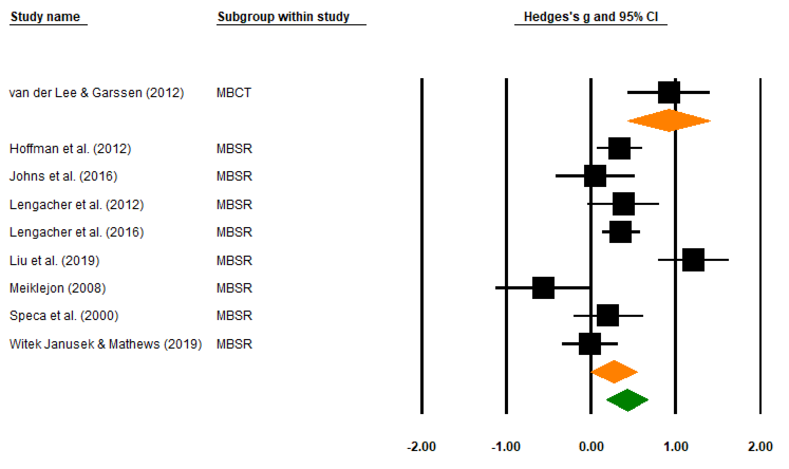

Supplement: S11 Fig — (TIF) [file pone.0269519.s017.tif]

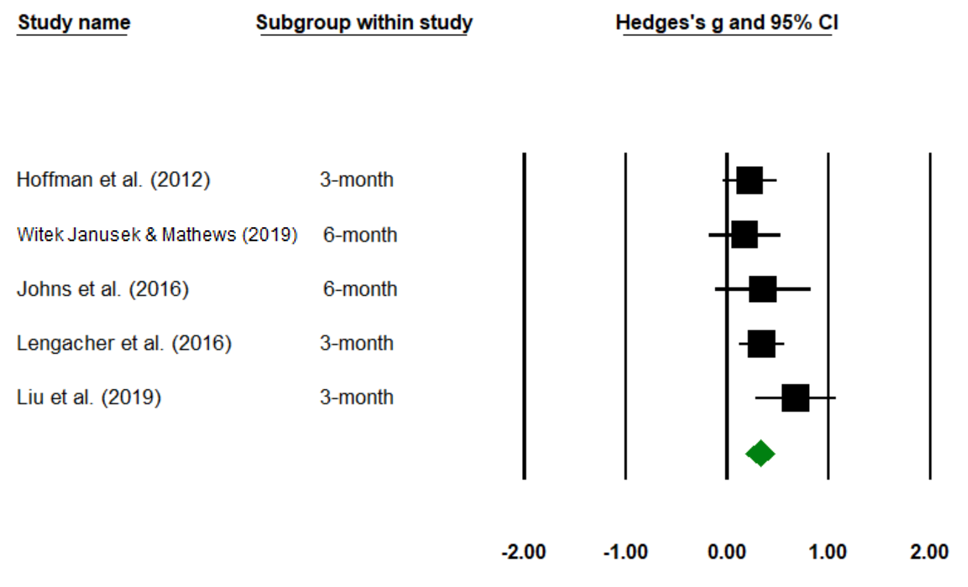

Supplement: S12 Fig — (TIF) [file pone.0269519.s018.tif]
