# Supplementary material for: The effects of mindfulness-based interventions on symptoms of depression, anxiety, and cancer-related fatigue in oncology patients: A systematic review and meta-analysis
Source: PLoS One. 2022 Jul 14;17(7):e0269519. doi: 10.1371/journal.pone.0269519 (PMC9282451; doi:10.1371/journal.pone.0269519)
Supplement: S1 Appendix — (DOCX) [file pone.0269519.s019.docx]

## **References in supporting information tables**

1. Birnie K, Garland SN, Carlson LE. Psychological benefits for cancer patients and their partners participating in mindfulness-based stress reduction (MBSR). *Psycho-Oncology.* 2010;19(9):1004-1009.
2. Carlson LE, Ursuliak Z, Goodey E, Angen M, Speca M. The effects of a mindfulness meditation-based stress reduction program on mood and symptoms of stress in cancer outpatients: 6-month follow-up. *Supportive Care in Cancer.* 2001;9(2):112.
3. Carlson LE, Speca M, Patel KD, Goodey E. Mindfulness-based stress reduction in relation to quality of life, mood, symptoms of stress, and immune parameters in breast and prostate cancer outpatients. *Psychosomatic medicine.* 2003;65(4):571-581.
4. Carlson LE, Garland SN. Impact of mindfulness-based stress reduction (MBSR) on sleep, mood, stress and fatigue symptoms in cancer outpatients. *International Journal of Behavioral Medicine.* 2005;12(4):278-285.
5. Chambers SK, Foley E, Galt E, Ferguson M, Clutton S. Mindfulness groups for men with advanced prostate cancer: a pilot study to assess feasibility and effectiveness and the role of peer support. *Supportive Care in Cancer.* 2012;20(6):1183.
6. Dobos G, Overhamm T, Büssing A, et al. Integrating mindfulness in supportive cancer care: a cohort study on a mindfulness-based day care clinic for cancer survivors. *Supportive Care in Cancer.* 2015;23(10):2945.
7. Garland SN, Carlson LE, Cook S, Lansdell L, Speca M. A non-randomized comparison of mindfulness-based stress reduction and healing arts programs for facilitating post-traumatic growth and spirituality in cancer outpatients. *Supportive Care in Cancer.* 2007;15(8):949.
8. Garland SN, Tamagawa R, Todd SC, Speca M, Carlson LE. Increased Mindfulness Is Related to Improved Stress and Mood Following Participation in a Mindfulness-Based Stress Reduction Program in Individuals With Cancer. 2013. In*.* Vol 122013:31-40.
9. Johns SA, Beck-Coon K, Stutz PV, et al. Mindfulness Training Supports Quality of Life and Advance Care Planning in Adults With Metastatic Cancer and Their Caregivers: Results of a Pilot Study. *American Journal of Hospice & Palliative Medicine.* 2020;37(2):88-99.
10. Kieviet-Stijnen A, Visser A, Garssen B, Hudig W. Mindfulness-based stress reduction training for oncology patients: Patients’ appraisal and changes in well-being. *Patient Education and Counseling.* 2008;72(3):436-442.
11. van den Hurk DGM, Schellekens MPJ, Molema J, Speckens AEM, van der Drift MA. Mindfulness-Based Stress Reduction for lung cancer patients and their partners: Results of a mixed methods pilot study. In*.* Vol 292015:652-660.
12. Johns SA, Brown LF, Beck-Coon K, Monahan PO, Tong Y, Kroenke K. Randomized controlled pilot study of mindfulness-based stress reduction for persistently fatigued cancer survivors. *Psycho-oncology.* 2015;24(8):885-893.
13. Johns S, Brown L, Beck-Coon K, et al. Randomized controlled pilot trial of mindfulness-based stress reduction compared to psychoeducational support for persistently fatigued breast and colorectal cancer survivors. *Supportive Care in Cancer.* 2016;24(10):4085-4096.
14. Kingston T, Collier S, Hevey D, et al. Mindfulness-based cognitive therapy for psycho-oncology patients: an exploratory study. *Irish journal of psychological medicine.* 2015;32(3):265-274.
15. Liu T, Zhang W, Xiao S, et al. Mindfulness-based stress reduction in patients with differentiated thyroid cancer receiving radioactive iodine therapy: a randomized controlled trial. *Cancer Management and Research.* 2019;ume 11:467-474.
16. van der Lee ML, Garssen B. Mindfulness-based cognitive therapy reduces chronic cancer-related fatigue: a treatment study. *Psycho-oncology.* 2012;21(3):264-272.
